# Systemic inflammation and subsequent risk of amyotrophic lateral sclerosis: Prospective cohort study

**DOI:** 10.1016/j.bbi.2023.07.026

**Published:** 2023-11

**Authors:** G. David Batty, Mika Kivimäki, Philipp Frank, Catharine R. Gale, Liam Wright

**Affiliations:** a Department of Epidemiology and Public Health, University College London, UK; b UCL Brain Sciences, University College London, UK; c Clinicum, Faculty of Medicine, University of Helsinki, Helsinki, Finland; d MRC Lifecourse Epidemiology Unit, University of Southampton, UK; e Lothian Birth Cohorts, Department of Psychology, University of Edinburgh, UK; f Centre for Longitudinal Studies, University College London, UK

## Abstract

**Background:**

While systemic inflammation has been implicated in the etiology of selected neurodegenerative disorders, its role in the development of amyotrophic lateral sclerosis (ALS), a condition with high case-fatality, is untested. Accordingly, we quantified the relationship of C-reactive protein (CRP), an acute-phase reactant and marker of systemic inflammation, with subsequent ALS occurrence.

**Methods:**

We used data from UK Biobank, a prospective cohort study of 502,649 participants who were aged 37 to 73 years when examined at research centers between 2006 and 2010. Venous blood was collected at baseline in the full cohort and assayed for CRP, and repeat measurement was made 3–7 years later in a representative subgroup (N = 14,514) enabling correction for regression dilution. ALS was ascertained via national hospitalization and mortality registries until 2021. We computed multivariable hazard ratios with accompanying 95% confidence intervals for log-transformed CRP expressed as standard deviation and tertiles.

**Results:**

In an analytical sample of 400,884 initially ALS-free individuals (218,203 women), a mean follow-up of 12 years gave rise to 231 hospitalizations and 223 deaths ascribed to ALS. After adjustment for covariates which included health behaviors, comorbidity, and socio-economic status, a one standard deviation higher log-CRP was associated with elevated rates of both ALS mortality (hazard ratios; 95% confidence intervals: 1.32; 1.13, 1.53) and hospitalizations (1.20; 1.00, 1.39). There was evidence of dose–response effects across tertiles of CRP for both outcomes (p for trend ≤ 0.05). Correction for regression dilution led to a strengthening of the relationship with CRP for both mortality (1.62; 1.27, 2.08) and hospitalizations (1.37; 1.05, 1.76).

**Conclusions:**

Higher levels of CRP, a blood-based biomarker widely captured in clinical practice, is associated with moderately increased future risk of amyotrophic lateral sclerosis.

## Highlights

• Higher CRP was associated with elevated rates of both ALS mortality and ALS hospitalizations.
• There was evidence of dose–response effects across CRP groups for both outcomes.
• Correction for regression dilution led to a strengthening of these relationships.

## 1 Key points

• *Question:* Is C-reactive protein (CRP), a marker of systemic inflammation widely used in clinical practice, associated with later risk of amyotrophic lateral sclerosis (ALS)?
• *Findings:* Following 12 years health surveillance in 400,884 individuals (218,203 women), after adjustment for covariates and correction for regression dilution, a dose–response relationship was apparent whereby a one standard deviation higher logCRP was associated with elevated rates of both mortality (hazard ratio; 95% confidence interval: 1.62; 1.27, 2.08) and hospitalizations (1.37; 1.05, 1.76) for ALS.
• *Meaning*: Elevated CRP may denote a higher likelihood of developing ALS.

## 2 Introduction

Amyotrophic lateral sclerosis (ALS), also known as motor neuron disease, involves the unabated degeneration of nerve cells responsible for voluntary muscle movement. With there being no effective treatment, death from respiratory failure typically occurs within 3 years of symptom emergence (Byrne et al., 2012, Abhinav et al., 2007). This brings into sharp focus the need for primary prevention research, yet a series of studies examining the role of modifiable risk factors, including biomarkers, have revealed disappointing results (Ingre et al., 2015, Al-Chalabi and Hardiman, 2013, Couratier et al., 2016, Oskarsson et al., 2018, Batty and Gale, 2021).

ALS is characterized by neuro-inflammation (Philips and Robberecht, 2011) and there is some evidence implicating C-reactive protein (CRP), an acute-phase reactant and widely used marker of systemic inflammation, in the disease process. In animal models, for instance, higher levels of CRP appear to increase the permeability of the blood–brain barrier, so triggering microglial activation (Hsuchou et al., 2012) leading to increased release of proinflammatory cytokines, neuroinflammation, and cell death in the brain (Beers et al., 2020). Evidence for a role of CRP in the etiology of ALS in humans is, however, largely circumstantial. While case-control studies have found that people with established ALS had higher CRP levels relative to the disease-free (Keizman et al., 2009, Ryberg et al., 2010), this could be a consequence of the condition rather than a cause. Surveillance of ALS patients reveals those with elevated systemic inflammation experienced a greater subsequent burden of disease severity, disability, and case fatality (Keizman et al., 2009, Sun et al., 2020, Lunetta et al., 2017), but there is a lack of studies of unaffected populations.

The absence of evidence from well-characterized prospective studies on the potential role of CRP as a risk indicator for the onset of ALS is in part ascribed to the rarity of this disorder, rendering many datasets insufficiently powered to yield sufficient cases to facilitate analyses. In UK Biobank (Sudlow et al., 2015), a prospective cohort study, a pre-morbid measurement of CRP was made in a cohort of 0.5 million members of the general population who subsequently underwent health surveillance via national registries. The repeat capturing of this inflammatory marker in this cohort also provides an unusual opportunity to address the impact of well-documented time-dependent fluctuations in CRP and all other exposures—referred to as regression dilution (Clarke et al., 1999)—when measurement on a single baseline occasion is likely to lead to an underestimation of the true magnitude of the relationship with ALS (Keogh and White, 2014).

## 3 Methods

UK Biobank is a UK-wide, on-going, closed, prospective cohort study. Described in detail elsewhere (Sudlow et al., 2015), between 2006 and 2010, 502,649 people aged 37 to 73 years attended 22 geographically disparate research clinics where they completed a questionnaire, underwent an interview, and took part in a medical examination. Ethical approval was obtained from the National Health Service National Research Ethics Service with all participants providing written consent. Using pseudonymized data, the present analyses did not require additional permissions. The present report follows STROBE guidelines for the presentation of original epidemiological research (von Elm et al., 2007).

### 3.1 Assessment of baseline characteristics

Non-fasting blood samples were collected from the participants at the baseline visit. High-sensitivity CRP levels were determined from serum using a standard immunoturbidimetry method on Beckman Coulter AU5800 apparatus. Assaying were also conducted for glycated hemoglobin (HbA1c), and high-density lipoprotein (LDL) cholesterol. Details regarding quality control and sample preparation methods have been described (Elliott and Peakman, 2008). Ethnicity was self-reported and categorized as White, Asian, Black, Chinese, Mixed, or other ethnic group (Lassale et al., 2020). Cigarette smoking and physical activity were measured using standard enquiries (Hamer et al., 2020). Self-reported physician diagnosis was collected for ALS, vascular or heart problems, diabetes, and cancer, and study members were asked if they had ever been under the care of a psychiatrist (Batty et al., 2016). The Townsend deprivation index, widely-used as an indicator of neighborhood socioeconomic circumstances and based on study member residential address (Batty et al., 2020), is continuously scored with higher values denoting greater deprivation.

Height and weight were measured directly and body mass index was calculated using the usual formulae (weight, kg/height^2^, m^2^) (Hamer et al., 2020). Forced expiratory volume in one second, a measure of pulmonary function, was quantified using spirometry with the best of three technically satisfactory exhalations used in our analyses. The best result from 3 trials on each hand using a hydraulic hand dynamometer provided a measure of handgrip strength, an indicator of overall musclature. Non-fasting venous blood was drawn, with assaying conducted at a dedicated central laboratory for glycated hemoglobin (HbA1c), and high-density lipoprotein (LDL) cholesterol (Hamer et al., 2020).

### 3.2 Resurvey of baseline characteristics

Depending on the baseline characteristic (risk factors, food intake, or imaging), up to 175,000 study members participated in resurveys at follow-up either by revisiting clinical research centres or by responding to the re-administration of on-line questionnaires (Bradbury et al., 2020, Frank et al., 2021, Rutter et al., n.d.). Following the baseline examination, in 2012–13 (3–7 years after baseline examination), a random sample of around 20,000 participants were invited back to research centres when most measurements and sample assays were repeated. Of these, 17,835 had their CRP levels reassessed, and 14,514 has viable data from both time points.

### 3.3 Ascertainment of amyotrophic lateral sclerosis during follow-up

Study participants were linked to the National Health Service’s (NHS) Central Registry which provided vital status data on study members and, where applicable, cause of death (Batty et al., 2019). Linkage was also made to hospital in-patients records via the NHS Hospital Episode Statistics, a registry of all hospitalizations in the UK (Abell et al., 2021). These registries provided comprehensive coverage of adverse health events for the entire study population until 23 March 2021 for mortality and 5 May 2021 for hospitalisation. Using both databases, ALS was denoted by the World Health Organization International Classification of Disease (version 10) code G122.

### 3.4 Statistical analyses

In all analyses, to address concerns regarding reverse causality – the notion that ALS might influence CRP (Cui et al., 2020) rather than the opposite – we excluded people who self-reported ALS at baseline medical examination and/or were hospitalized with the disease before study induction (N = 85). Additionally, to capture study members with potentially subclinical (undiagnosed) ALS, we left-censored study members such that those who were hospitalized for, or died from, the condition within the first 3 years of baseline were also excluded (N = 61). Lastly, study members with a CRP value ≥ 10.0 mg/L, indicative of an acute infection, were also excluded (Akbaraly et al., 2013). This resulted in analytical sample of 400,884 (218,203 women) with full data on CRP, covariates, and ALS outcomes.

To summarize the association between CRP and ALS, we used Cox proportional hazards regression to compute hazard ratios with accompanying 95% confidence intervals (Cox, 1972). In these analyses, calendar period was the time scale and study members were censored at date of hospitalization or death from ALS, or end of follow-up – whichever came first. CRP was utilized as a standardized, log-transformed linear term (mean = 0, SD = 1) and as tertiles. To examine potential non-linearity in the association between CRP levels and rates of ALS, we included CRP in models using three natural cubic splines (Perperoglou et al., 2019). We performed these analyses separately for ALS hospitalisations and ALS deaths.

Regression dilution bias is the attenuation of an estimated relationship between an exposure and an outcome towards the null owing to random measurement error in that exposure (Clarke et al., 1999). To address it, we ran models including a dilution corrected value for CRP following a univariate regression calibration procedure (Keogh and White, 2014). This involved regressing the second measure on the first and using the model predictions to calculate corrected CRP values for the full analytic sample. Using this derived value for CRP in the Cox model, confidence intervals were estimated via bootstrapping (1,000 replications). All analyses were performed using R version 4.1.2 (R Core Team R, 2021). The syntax used in the present analyses is available on-line (https://osf.io/hzsc3/).

## 4 Results

In Table 1 we show baseline data according to tertiles of CRP. Less favorable levels of physiological, social, behavioral, and co-morbid characteristics were universally apparent in people with higher CRP. For instance, people with higher levels of CRP tended to have higher body mass index, lower lung function, and lower socio-economic position. Study members with higher CRP also had a greater prevalence of cigarette smoking, physical inactivity, and an array of chronic illnesses, including vascular disease, cancer, diabetes, and mental illness.

**Table 1:** Baseline characteristics of study members according to C-reactive protein levels at baseline in UK Biobank (2006–2020).

	Tertile 1 (<=0.78 mg/L)	Tertile 2 (0.79–1.88 mg/L)	Tertile 3 (1.89–9.99 mg/L)
Number of people (women)	134,572 (73,296)	132,984 (68,479)	133,328 (76,428)
C-Reactive Protein, mg/L	0.47 (0.18)	1.27 (0.31)	3.85 (1.86)
Age, yr	54.99 (8.16)	56.74 (8.03)	57.29 (7.92)
Glycated hemoglobin, mmol/mol	34.75 (5.18)	35.72 (5.94)	37.09 (7.47)
Grip strength, kg	33.79 (11.18)	33.38 (11.33)	31.58 (11.19)
High-density lipoprotein cholesterol, mmol/L	1.55 (0.39)	1.45 (0.38)	1.38 (0.35)
Forced Expiratory Volume in 1 sec, L	3.03 (0.80)	2.88 (0.78)	2.69 (0.76)
Body mass index, kg/m^2^	24.96 (3.43)	27.16 (3.89)	29.45 (5.00)
Neighborhood deprivation score	−1.58 (2.94)	−1.49 (2.99)	−1.15 (3.14)
Non-White, N (%)	6,767 (5.03)	6,391 (4.81)	6,972 (5.23)
Current cigarette smoker, N (%)	10,443 (7.76)	12,307 (9.25)	17,028 (12.77)
Low physical activity, N (%)	4,893 (3.64)	6,843 (5.15)	11,142 (8.36)
Vascular disease, N (%)	28,251 (20.99)	37,638 (28.30)	46,804 (35.10)
Diabetes, N (%)	4,708 (3.50)	6,039 (4.54)	8,163 (6.12)
Cancer, N (%)	8,569 (6.37)	9,527 (7.16)	10,785 (8.09)
Psychiatric consultation, N (%)	13,769 (10.23)	13,900 (10.45)	16,206 (12.15)

Results are mean (SD) unless otherwise stated. Levels of all baseline characteristics were statistically heterogeneous across tertiles of CRP (p-value p < 0.0001).

**Fig. 1:**
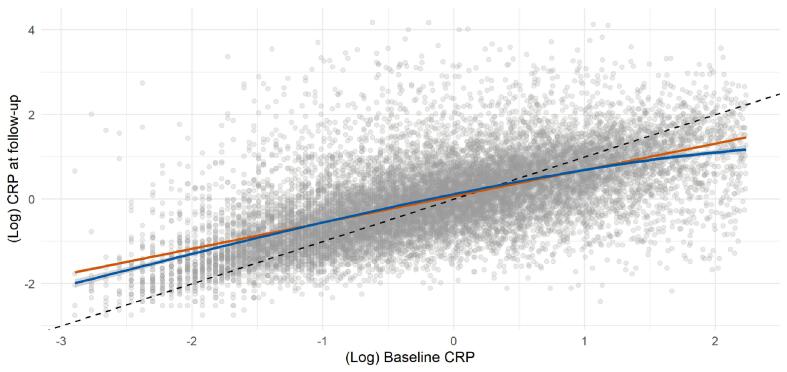
Scatterplot of CRP from baseline (2006–2010) and follow-up (2012–2013) (N = 14,514).

Mortality surveillance over an average of 11.88 years (range 0.01 – 14.26) in 400,884 people resulted in 223 deaths attributed to ALS, and in these analyses higher baseline CRP levels were related to a higher subsequent risk of this disorder (Table 2 and supplemental Fig. 1). Thus, after adjustment for multiple covariates, the highest tertile of systemic inflammation was associated with a 75% increase in the risk of death from ALS (hazard ratio; 95% confidence interval: 1.75; 1.21, 2.53). A dose–response relationship was also apparent (p-value for trend < 0.001) such that the central tertile of CRP carried intermediate risk (1.46; 1.02, 2.08). When expressed per standard deviation increase in log of baseline CRP, there was a 32% increase in the risk of ALS (1.32; 1.13, 1.53). When we utilized resurvey data on CRP levels to examine the impact of repression dilution (correlation coefficient for survey-resurvey CRP was 0.64 [N = 14,514, p-value < 0.001; Fig. 1]), the magnitude of relative risk per SD approximately doubled with this correction (1.62; 1.27, 2.08).

**Table 2:** Association of C-reactive protein at baseline (2006–2010) with ALS risk at follow-up (2006–2010 to 2021) in UK Biobank (N = 400,884).

		Tertile 1 (<=0.78 mg/L)	Tertile 2 (0.79–1.88 mg/L)	Tertile 3 (1.89–9.99 mg/L)	p-value for trend	Per 1 SD increase (observed)	Per 1 SD increase (dilution corrected)
Death	N cases / N at risk	53 / 134,572	80 / 132,984	90 / 133,328			
	Age- & sex-adjusted	1.00 (ref)	1.35 (0.95, 1.91)	1.49 (1.06, 2.10)	0.02	1.21 (1.06, 1.39)	1.38 (1.11, 1.69)
	Multiply-Adjusted	1.00 (ref)	1.46 (1.02, 2.08)	1.75 (1.21, 2.53)	< 0.001	1.32 (1.13, 1.53)	1.62 (1.27, 2.08)
Hospitalization	N cases / N at risk	62 / 134,572	79 / 132,984	90 / 133,328			
	Age- & sex-adjusted	1.00 (ref)	1.16 (0.83, 1.61)	1.30 (0.94, 1.80)	0.11	1.14 (0.99, 1.30)	1.24 (1.00, 1.53)
	Multiply-Adjusted	1.00 (ref)	1.22 (0.87, 1.71)	1.43 (1.00, 2.04)	0.05	1.20 (1.03, 1.39)	1.37 (1.05, 1.76)

Multiple adjustment is adjustment for: age, sex, ethnicity, smoking status, physical activity, body mass index, co-morbidity (vascular disease, diabetes, cancer, mental illness), lung function, and Townsend deprivation score.

A mean follow-up of 12.0 years (range 0.01–14.4) in 400,884 individuals gave rise to 231 hospitalizations for ALS (117 women). The pattern of association for hospitalization and CRP was similar to that seen for mortality but effect estimates were typically of lower magnitude and did not always achieve statistical significance at conventional levels (Table 2 and supplemental Fig. 1). Again, a strengthening of the CRP–ALS association was apparent after taking into account regression dilution bias. Thus, the multiply-adjusted hazard ratio per SD increase in CRP increased from 1.20 (1.03, 1.39) to 1.37 (1.05, 1.76).

**Fig. 2:**
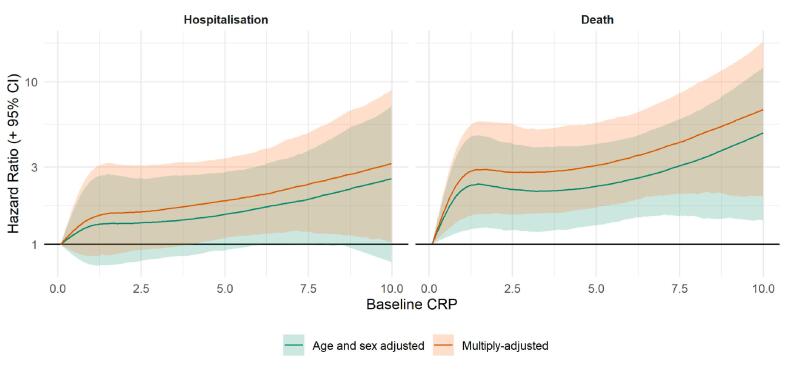
Association of C-reactive protein at baseline (2006–2010) with ALS risk at follow-up (2006–2010 to 2021) in UK Biobank (N = 400,884). Multiple adjustment is adjustment for: age, sex, ethnicity, smoking status, physical activity, body mass index, co-morbidity (vascular disease, diabetes, cancer, mental illness), lung function, and Townsend deprivation score.

We conducted a series of sensitivity analyses. First, splines allowed us to scrutinize these apparent dose–response effects for CRP in relation to ALS (Fig. 2) by searching for any inflection that would have been hidden by analyses of the three-group categorization of CRP. There was a suggestion that the steepest elevation in risk was apparent at lower levels of CRP with a less pronounced increase thereafter. The shape of the association was broadly similar for both deaths and hospitalizations. Second, just as there will be variation in CRP values over time, levels of the covariates in the present study are similarly time-dependent. Correcting for time-variance in covariates, in addition to CRP, however, revealed very similar results to those apparent when correcting for CRP alone: hospitalization for ALS (a one SD increase in CRP: 1.37; 1.05, 1.80) and mortality from ALS (1.64; 1.24, 2.10). Third, we ran further survival analyses where we added HbA1c, HDL cholesterol, and grip strength, available in a subset of the study members, to our existing multivariable model. After these more extensive adjustments, our results were again essentially unchanged for both ALS hospitalization (a one SD increase in CRP: 1.20; 1.03, 1.41) and death (1.30; 1.10, 1.52).

## 5 Discussion

Our main findings were that, in a population initially apparently free of ALS, higher baseline CRP was moderately strongly related to a subsequently elevated risk of developing this neurodegenerative disorder. This association was somewhat stronger for ALS mortality relative to hospitalization; strengthened by adjustment for multiple confounding factors; and of higher magnitude after correction for regression dilution. Although confidence intervals are largely overlapping, the slightly stronger association of CRP with ALS mortality compared to ALS hospitalizations is biologically plausible--the duration of follow-up is the same for both endpoints and ALS death is later in the disease process when the manifestation of the illness is most severe. The two non-genetic factors most closely linked to the development of ALS – age and sex (Armon, 2003) – were recapitulated here. Thus, people who were older (hazard ratio; 95% confidence interval per decade increase: 2.08; 1.72, 2.50) and male (1.16; 0.90, 1.50) experienced elevated rates of hospitalization (corresponding results for death from ALS were 1.20; 0.92, 1.56; and 2.42; 1.98, 2.95). This gives us some confidence in the novel results for CRP.

### 5.1 Comparison with other studies

Elevated levels of systemic inflammation have been shown to be prospectively associated with higher rates of other neurodegenerative disorders such as dementia (Kuo et al., 2005) and Parkinson’s disease (Qiu et al., 2019). With this study being, to our knowledge, the first to specifically examine the role of pre-morbid CRP as a potential risk factor for ALS occurrence, direct comparison of our results with other data is not possible. In a study most closely resembling our own, however, haptoglobin, a little-utilized marker of systemic inflammation, was not related to the development of ALS 15 years later (Yazdani et al., 2019). Relatedly, in vitro and animal work suggests that the actions of the neuroinflammation-promoting enzyme, cyclooxygenase-2, are inhibited by non-steroidal anti-inflammatory drugs (Carty et al., 2011, Klegeris and McGeer, 2002). In cohort analyses, however, regular use of nonsteroidal anti-inflammatory medication was not associated with the development of ALS (Popat et al., 2007, Fondell et al., 2012).

### 5.2 Mechanisms of effect

That the association between CRP and ALS was robust to the adjustment of potential confounding and mediating factors raises the suggestion of a direct effect. The observation that the steepest elevation in risk was apparent at lower levels of CRP with a less pronounced increase thereafter suggests the mechanism relates to low-grade systemic inflammation. One possibility is that even modestly raised of CRP denotes inflammation that increases the permeability of the blood–brain barrier, so triggering microglial activation (Hsuchou et al., 2012) leading to enhanced release of proinflammatory cytokines, neuroinflammation, and cell death in the brain (Beers and Appel, 2019). CRP is, however, a systemic marker of inflammation; that is, it does not provide a diagnosis of any specific condition and should be only used as a basis for further clinical testing. Additional large-scale research is needed to evaluate the link, if any, of a wider set of inflammation-related blood-based biomarkers implicated in the aetiology of ALS, including misfolded and aggregated proteins related to pathogen-associated molecular patterns (PAMPs) and damage-associated molecular patterns (DAMPs), circulating heat-shock proteins (HSPs), viral and bacterial antigens, oxidised lipids (e.g., the Toll-like receptor (TLR) proteins, C-type lectins and oxidized lipoprotein detectors) and soluble Triggering Receptor Expressed on Myeloid Cells 2 (TREM2) (Stephenson et al., 2018, Appel et al., 2021).

### 5.3 Study strengths and limitations

The strengths of the present study include its novelty; the use of recapture data on CRP to examine regression dilution bias; its scale, which facilitates the accumulation of a sufficiently high number of cases for analyses of a rare neurodegenerative disorder alongside left-censoring to take into account reverse causality; and the well-characterized nature of the study participants which facilitates adjustment for multiple confounding factors.

Inevitably, however, our work has its weaknesses. First, the present study sample comprises only the 5.5% of the target population (Sudlow et al., 2015). As has been demonstrated (Fry et al., 2017, Batty et al., 2020), the data material is therefore inappropriate for estimation of risk factor or disease prevalence and for event incidence, including for ALS. These observations do not, however, seem to influence reproducibility of the association of established risk factors for important health outcomes such as vascular disease, selected cancers, and suicide (Batty et al., 2020). We think the same reasoning can be applied to the present analyses for ALS. Second, we did not have data on other markers of systemic inflammation such as interleukin 6 with which to draw comparison with the present results for CRP. Lastly, the outcomes herein represent advanced ALS and it is unknown if CRP is related to ALS at earlier stages of disease progression.

In conclusion, in the present study, higher levels of pre-morbid CRP, a modifiable inflammatory marker commonly captured in clinical practice, appeared to be associated with elevated risk of incident ALS. These observational results warrant further testing, as do the mechanisms linking systemic inflammation to ALS pathology.

Access to data

Data from UK Biobank are available upon application (https://www.ukbiobank.ac.uk/). Part of the present research has been conducted using the UK Biobank Resource under Application 10279.

## Funding

GDB is supported by the UK Medical Research Council (MR/P023444/1; MR/X003434/1) and the US National Institute on Aging (1R56AG052519-01; 1R01AG052519-01A1), and MK by the Wellcome Trust (221854/Z/20/Z), the UK Medical Research Council (MR/S011676/1), the US National Institute on Aging (R01AG056477), and the Academy of Finland (350426). These funders did not provide direct financial or material support for the work, and had no role in study design, data collection, data analysis, data interpretation, or report preparation.

## Declaration of Competing Interest

The authors declare that they have no known competing financial interests or personal relationships that could have appeared to influence the work reported in this paper.

## Data Availability

The authors do not have permission to share data.
