# Supplementary material for: Systemic inflammation and subsequent risk of amyotrophic lateral sclerosis: Prospective cohort study
Source: Brain Behav Immun. 2023 Nov;114:46–51. doi: 10.1016/j.bbi.2023.07.026 (PMC10937260; doi:10.1016/j.bbi.2023.07.026)
Supplement: Supplementary data 1 [file mmc1.docx]

**Supplemental figures**

**Batty GD, Kivimäki M, Frank P, Gale CR, Wright L. Systemic inflammation and amyotrophic lateral sclerosis: prospective cohort study**

Supplemental figure 1. Association of C-reactive protein at baseline (2006-2010) with ALS risk at follow-up (2006-2010 to 2021) in UK Biobank (N=400,884)

Supplemental figure 2. Association of C-reactive protein at baseline (2006-2010) with ALS risk at follow-up (2006-2010 to 2021) in UK Biobank (N=400,884) – without and with correction for regression dilution bias

**Supplemental figure 1. Association of C-reactive protein at baseline (2006-2010)**

**with ALS risk at follow-up (2006-2010 to 2021) in UK Biobank (N=400,884)**

**
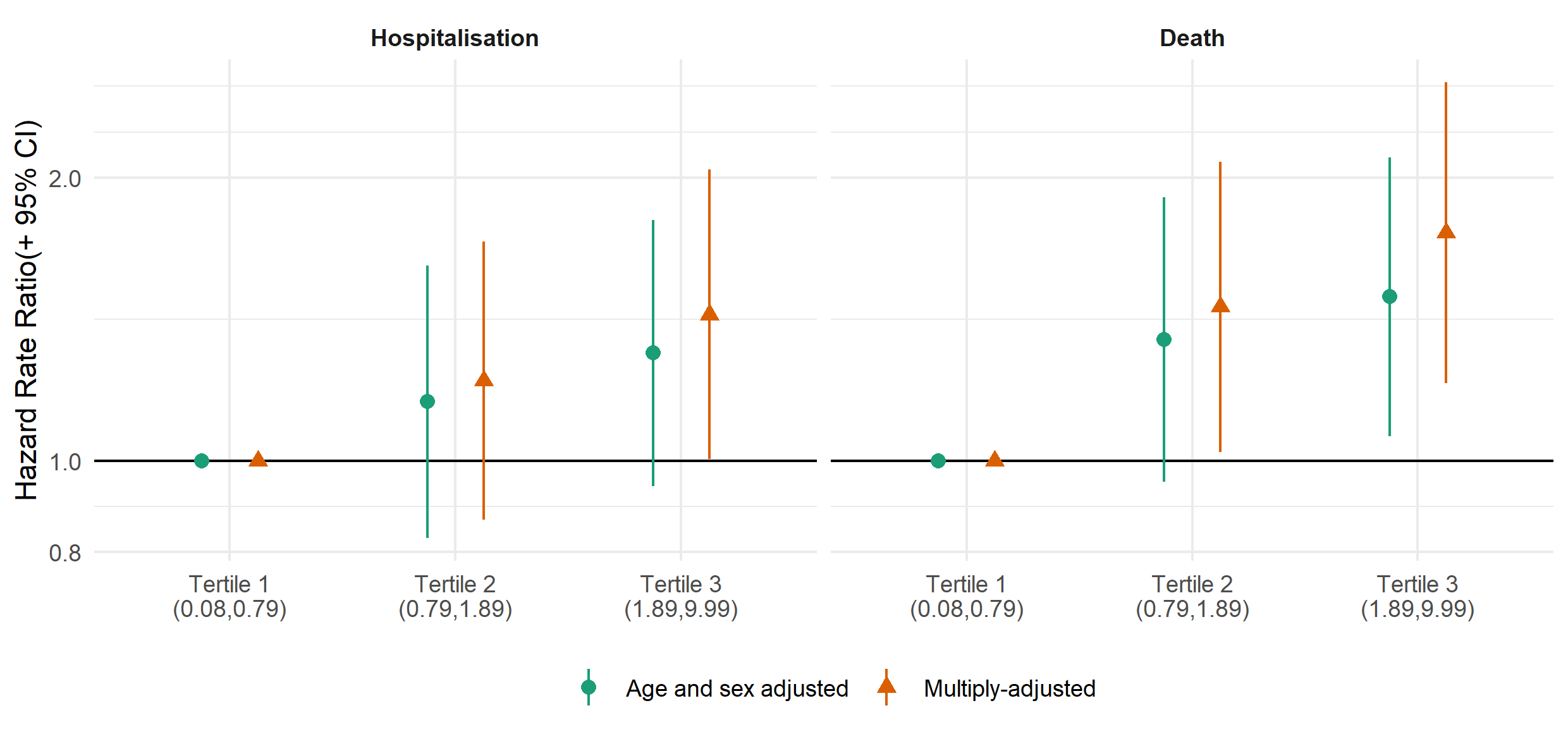
**

Multiple adjustment is adjustment for: age, sex, ethnicity, smoking status, physical activity, body mass index,

co-morbidity (vascular disease, diabetes, cancer, mental illness), lung function, and Townsend deprivation score

**Supplemental figure 2. Association of C-reactive protein at baseline (2006-2010)**

**with ALS risk at follow-up (2006-2010 to 2021) in UK Biobank (N=400,884) –**

**without and with correction for regression dilution bias**

**
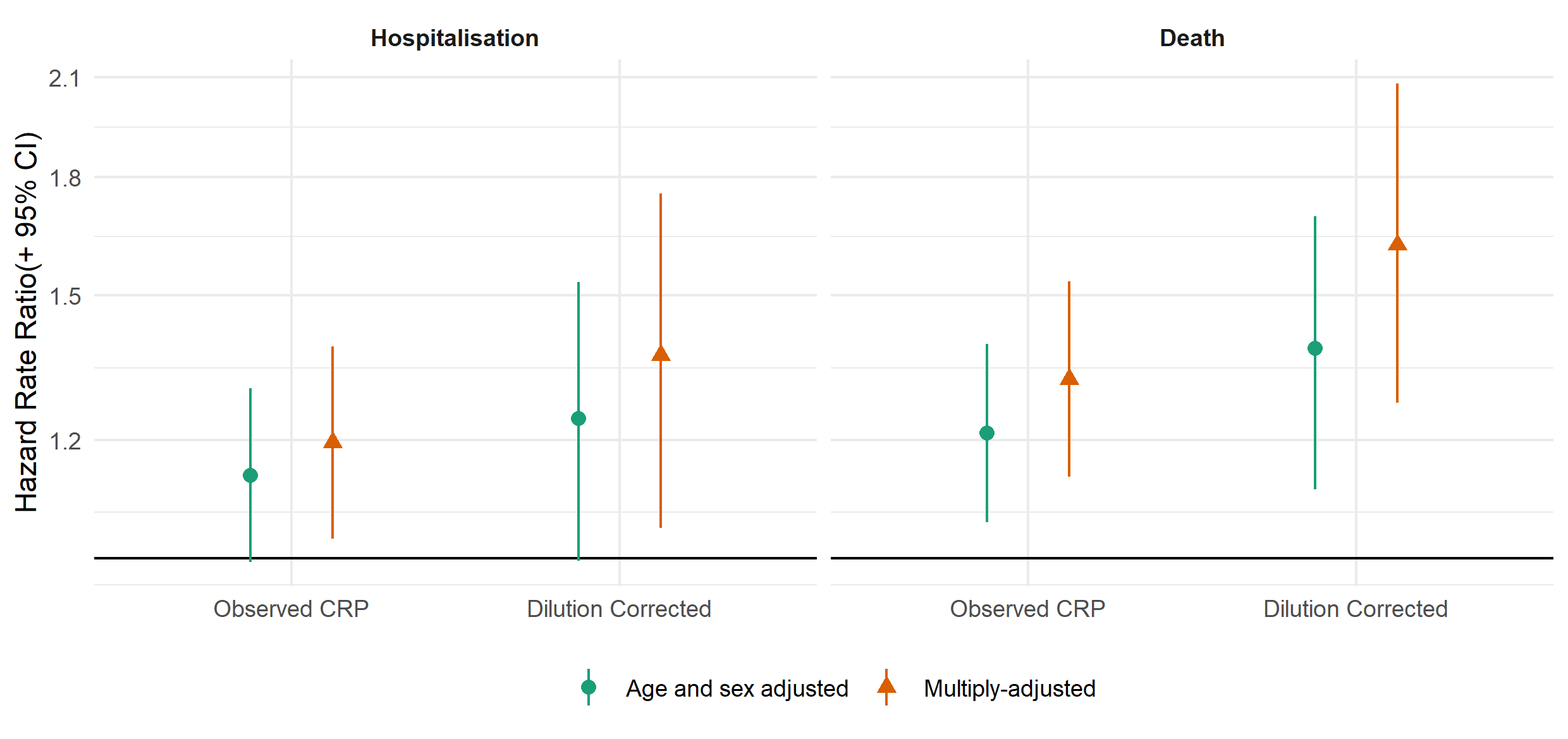
**

Multiple adjustment is adjustment for: age, sex, ethnicity, smoking status, physical activity, body mass index,

co-morbidity (vascular disease, diabetes, cancer, mental illness), lung function, and Townsend deprivation score
